# Measures of healthcare decision-making ability in cognitive aging: a scoping review from the Advancing Reliable Measurement in Cognitive Aging and Decision-Making Ability (ARMCADA) research initiative

**DOI:** 10.3389/fpubh.2025.1582764

**Published:** 2025-06-12

**Authors:** Molly A. Mather, Berivan Ece, Tatiana Karpouzian-Rogers, Emily H. Ho, Patricia Bucko, Elizabeth M. Dworak, Miriam A. Novack, Sarah Pila, Zahra Hosseinian, Janel Hanmer, Richard C. Gershon, Sandra Weintraub

**Affiliations:** ^1^Mesulam Center for Cognitive Neurology and Alzheimer’s Disease, Northwestern University Feinberg School of Medicine, Chicago, IL, United States; ^2^Department of Psychiatry and Behavioral Sciences, Northwestern University Feinberg School of Medicine, Chicago, IL, United States; ^3^Department of Medical Social Sciences, Northwestern University Feinberg School of Medicine, Chicago, IL, United States; ^4^Department of Medicine, University of Pittsburgh, Pittsburgh, PA, United States

**Keywords:** healthcare decision, decision-making capacity, patient decision-making, health outcomes, capacity assessment instruments, decision-making assessment

## Abstract

**Introduction:**

Declines in decision-making (DM) ability are often observed with increasing age and pose significant risk for negative health, financial, and functional outcomes. The Advancing Reliable Measurement in Cognitive Aging and Decision-making Ability (ARMCADA) research initiative aims to improve measurement of DM ability in aging to facilitate early detection of cognitive and functional decline. This scoping review summarizes the extant literature on DM measures in aging, focusing specifically on measures relevant to healthcare decision-making (HCDM).

**Methods:**

We identified articles published between 2018 and 2023 using keywords related to DM abilities in aging populations. Titles and abstracts were first reviewed by two trained reviewers, followed by full-text review and extraction. Results of the current scoping review are reported in adherence to PRISMA-ScR guidelines.

**Results:**

The scoping review identified 16,286 articles across multiple domains of decision-making, 705 of which met criteria for extraction, and 246 of which were related to healthcare decision-making. There were 86 unique measures across these articles, and 18 of these measures directly targeted decision-making ability. Most measures were administered to clinical groups in English and in-person with a trained examiner. Measures of healthcare DM ability tended to consist of semi-structured interviews or performance-based items, though there were also several self-report measures.

**Discussion:**

The most commonly used measures to assess HCDM ability require trained administration of a semi-structured interview to assess ability to reason about health-related scenarios and are often time-intensive. Creation of a streamlined, standardized measure to assess HCDM ability will benefit both research and clinical care for the aging population.

## 1 Introduction

The ability to make effective decisions about one’s health has long been recognized as an important aspect of overall wellbeing, particularly as individuals age (e.g., (1)). As the global population continues to rapidly age (e.g., (2)) and medical decisions grow increasingly complex, reliable and efficient measures of even minor declines in decision-making (DM) ability have become critical. However, assessing DM ability has historically been challenging due to inconsistent definitions of decision capacity, low inter-rater reliability, and differing practices among assessors (e.g., (3)).

Healthcare-related decision making (HCDM) refers to the process by which individuals make choices and judgments regarding their own health and health-related matters. Amidst a highly complex healthcare system, individuals are tasked with navigating an increasing number of decisions related to access to and use of healthcare resources, including choices about specific healthcare insurance plans and the feasibility of access to care (4). Whereas ongoing advancements in the treatment of acute and chronic illness have vastly improved overall survival and health, they also contribute to decision complexity. For example, there is no clear correct answer when deciding whether to move forward with a treatment that has a certain percent chance of extending survival but carries a significant chance of side effects that would meaningfully impact patient quality of life. Indeed, health-related choices are often inherently value-based (e.g., (5)), and it is necessary to consider individual values and priorities that may lead to differing decisions when assessing HDCM ability.

HCDM is often conceptualized using a four-component model (6). Under the Grisso and Appelbaum model (6), a patient can demonstrate capacity to make a specific healthcare decision by exhibiting the ability to (1) understand the situation and relevant options, (2) appreciate how a situation applies to them, (3) reason about which choice is most appropriate, and (4) express a choice. Whereas many persons with cognitive decline may be able to express a choice and provide reasons for that choice (7), the capacity to fully understand and appreciate the various features of a choice is often more complex and more likely to be susceptible to even early stages of cognitive decline. It is crucial for measures of healthcare decision-making (HCDM) ability to be adequately sensitive to pick up on subtle changes in understanding, appreciating, and even reasoning about healthcare choices.

The current scoping review is part of the larger Advancing Reliable Measurement in Cognitive Aging and Decision-making Ability (ARMCADA) research initiative, which aims to develop and validate a multidomain DM battery for research and clinical use. To inform this effort, we conducted a scoping review of measures of DM across multiple domains that have been used in adults aged 45 + and with a variety of participant populations (e.g., healthy controls, persons with cognitive impairment, persons with chronic or terminal illnesses). Inclusion of participants aged 45 and above, rather than typical cut offs of 50–60 years old, allows for investigation of the earliest signs of decline in DM associated with age and neurodegenerative diseases. The goal of the larger scoping review was to identify the most frequently used measures of DM in recent years to establish limitations of currently available approaches to measuring DM ability; the current paper focuses solely on HCDM.

## 2 Methods

The scoping review was guided by the framework of Arksey and O’Malley (8) with methodology and results reported in line with the PRISMA Extension for Scoping Reviews (PRISMA-ScR; (9)). A preliminary search of MEDLINE, the Cochrane Database of Systematic Reviews, and JBI Evidence Synthesis identified no existing or ongoing systematic or scoping reviews on this topic and with this specific participant population (e.g., individuals aged 45 and older) prior to starting our review.

### 2.1 Protocol and registration

The HCDM domain review was conducted as part of a larger comprehensive scoping review (see (10) for review protocol). This study did not involve human subjects research, and therefore full institutional review board approval was not required (Northwestern University STU00220334).

### 2.2 Search strategy and eligibility criteria

The initial database search for the multi-domain scoping review included terms broadly related to DM and aging (e.g., “decision making,” “decision capacity,” “decisional impairment”; see (10) for full search terms across domains), as well as domain-specific terms for each included domain (see Table 1 for terms specific to healthcare). Databases included Embase (Elsevier; 10,114 results), MEDLINE (Ovid; 4,528 results), PsycINFO (EbscoHost; 3,615 results), Cochrane Library (Wiley; 831 results), Web of Science (Clarivate), and Scopus (Elsevier; 5,448 results). The search strategy was developed with the help of a medical research librarian at Northwestern University’s Galter Health Sciences Library.

**Table 1:** Example search terms for healthcare decision-making.

	Healthcare specific search terms
General search terms	‘patient decision making’
‘choice behavior’	‘healthcare decision’
‘choice making’	‘health care decision’
‘decisional impairment’	‘health related decision’
‘decision process’	‘medical decision’
‘decision making task’	‘health care planning’
‘decision quality’	‘health literacy’
‘decision capacity’	‘informed consent’
‘geriatrics’	‘medical information’
‘geriatrician’	‘attitude to health’
	‘health belief model’
‘elder abuse’	‘aged patient’

We identified articles published between January 1st, 2018 and November 6th, 2023 using keywords related to DM abilities across domains. This scoping review focused on identifying measures used to assess decision-making ability in aging populations, and we limited our search to the prior 5 years to ensure that we captured measures that are being contemporaneously used in research and clinical settings and that are likely to be adaptable to digital formats. Articles were assigned to relevant domains by trained reviewers during the full-text review and extraction stages. The focus of the current project was to identify measures of individual DM abilities, so articles that only included measures of clinical or shared DM, decision aids, or low-level cognitive abilities (e.g., lexical decision tasks) were excluded.

### 2.3 Screening, data extraction, and synthesis

Once the initial search was completed, the articles were screened in Covidence (11), a software management system for scoping reviews, using the inclusion and exclusion criteria provided in Table 2. This was carried out in three stages: (1) title and abstract screening, (2) full-text review, and (3) full-text extraction and synthesis. For each stage, all reviewers were trained on sample articles as a group and then completed individual reviews on a subset of sample articles. After their individual reviews were assessed and approved by the domain scientists, reviewers proceeded to the screening process.

**Table 2:** Inclusion and exclusion criteria for overall ARMCADA scoping review.

Factor	Inclusion criteria	Exclusion criteria
Population	Adults over age 45 The assessment was conducted with at least one group of individuals over 45 years Age range includes participants aged 45 and over	Adults ≤45 years old
Study Characteristics	The study mentions at least one assessment of one or more of the target domains The domain of interest is an outcome assessed by the study.	Single-subject research/Case studies. Focus group Review articles Narrative reviews Gray literature Conference Proceedings Books and/or book chapters Commentaries Preprints Other non-research publications
Other	Language of measures: All languages Location: All geographical locations	Article published in a language other than English and no English translation available Articles that only measure shared decision-making Articles that only measure decision aids

#### 2.3.1 Title and abstract screening

Covidence was utilized for the title and abstract screening. In the first stage of the review, each article was screened by two trained reviewers independently focusing on the title and abstract only. Conflicts between reviewers were resolved by another trained reviewer or doctoral level project scientist. This first stage of screening was conducted from November 10th to December 8th, 2023. The articles included were then entered into the full-text review stage.

#### 2.3.2 Full-text review

The full text review was also conducted using Covidence. Each article’s full text was independently assessed by two trained reviewers to confirm inclusion/exclusion using the eligibility criteria (see Table 2). Conflicts were resolved by a third expert reviewer. Full text review of articles was conducted from December 8th to December 22nd, 2023.

#### 2.3.3 Full-text extraction and synthesis

The extraction phase was completed via Qualtrics.^1^ Trained reviewers extracted data from articles that were not excluded during title and abstract review or during full-text review. Extracted variables included the age of the sample, DM domain(s) assessed, and various features of the included measures (e.g., language of administration, duration, required technology or materials, mode of administration). Once data extraction was completed on January 31st, 2024, data from Qualtrics was exported to Excel for preliminary categorization (e.g., assignment to relevant DM domains).

## 3 Results

### 3.1 Search results

**Figure 1:**
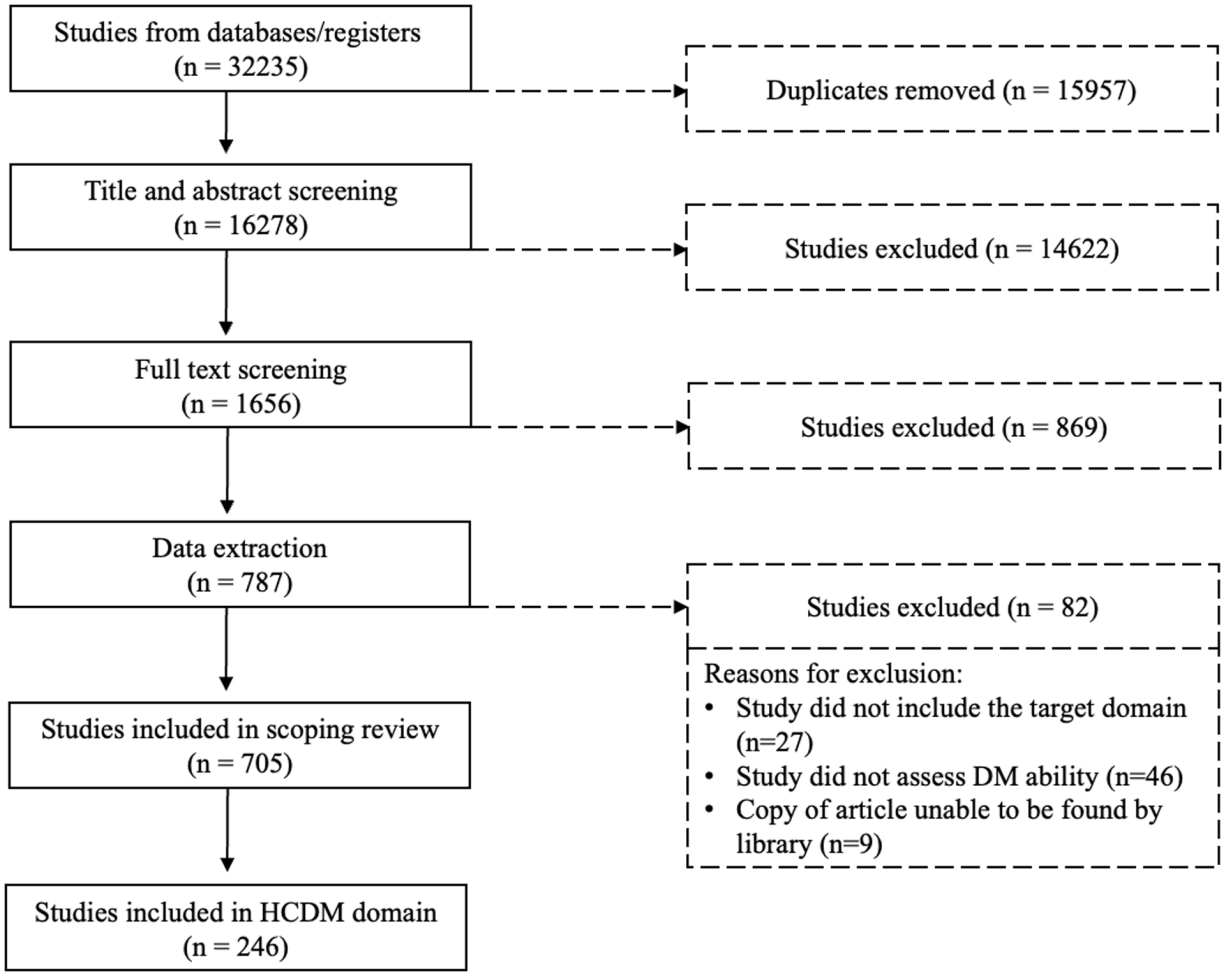
PRISMA flowchart.

The initial search yielded 32,235 records based on the search strategy. Covidence was used to remove 15,957 duplicate records and screen the remaining 16,278 articles, first by title and abstract (14,622 excluded) and then by full-text screening (869 excluded). The remaining 787 were then moved to the extraction phase; 82 articles were excluded during extraction, resulting in a final set of 705 articles (see Figure 1 for details). Of these 705 articles, 246 focused on healthcare decision-making, broadly defined, in persons aged 45 and older. Out of the 246 articles that discussed healthcare decision-making, we excluded an additional 57 articles as they included only measures that fell under other DM domains (e.g., Financial DM or Functional Outcomes) rather than HCDM. Thus, 189 articles included at least one measure directly assessing HCDM or other healthcare-related topics (e.g., decision style, treatment satisfaction, health behaviors). There were 86 unique measures included across the 189 articles, 18 of which were direct measures of healthcare DM ability, ranging from semi-structured interviews and vignettes to self-report questionnaires (see Table 3). The remaining measures (see Supplementary Table 1) focused on decision-making behaviors (*n* = 11; e.g., decision-making style, extent of deliberation before making a decision), feelings and attitudes about decisions (*n* = 14; e.g., decisional conflict, decisional regret, satisfaction with decisions), health behaviors and outcomes (*n* = 31; e.g., treatment adherence, health-related quality of life, health literacy, motivation for treatment), capacity to consent to research (*n* = 4), shared decision-making (*n* = 6), proxy decision-making (*n* = 1), and effectiveness of a decision aid (*n* = 1), all of which fall outside of the scope of this review.

**Table 3:** Characteristics of measures assessing HCDM ability.

Measure	# Citations in scoping review	Citing articles	Original citation	Age ranges covered	Clinical groups	Modality	In-person vs. remote	Examiner- vs. Self-administered
MacArthur Competence Assessment Tool for Treatment (MacCAT-T)	22 (1 modified version)	Aki et al. (36); Carabellese et al. (37); Chang and Bourgeois (38); Curley et al. (39); Curley et al. (40); Kato et al. (41); Kolva et al. (42); Koukopoulos et al. (43); Loughran et al. (44); Mandarelli et al. (45); Murphy et al. (46); Murphy et al. (47); Nystazaki et al. (48); Ogawa et al. (49); Olie et al. (50); Oshima et al. (51); Parmigiani et al. (52); Poth et al. (53); Santos et al. (54); Spataro and La Bella (55); Sugawara et al. (56); Turner et al. (57)	Grisso and Applebaum (6)	18–85+	Mild cognitive impairment; Alzheimer’s disease dementia; Dementia; Schizophrenia spectrum disorders; Psychiatric inpatients; Suicide attempters; Terminal cancer; Bipolar disorder; Major depressive disorder; Lung cancer; Amyotrophic lateral sclerosis (ALS)	Semi-structured interview w/vignettes	In-person	Administered by examiner
Decision Making Competence Assessment Tool (12-item Assessment)	12	Glover et al. (58); Han et al. (59); Kapasi et al. (60); Lamar et al. (61); McSorley et al. (62); Stewart et al. (63); Stewart et al. (64); Stewart et al. (65); Weissberger et al. (66); Wilson et al. (67); Yu et al. (68); Yu et al. (69)	Finucane and Gullion (15)	45–85+	Alzheimer’s disease dementia; Common chronic conditions of aging	Performance-based	In-person	Administered by examiner
The Decision Self-Efficacy Scale (DSE)	8 (1 modified version)	Bakhit et al. (70); Cuypers et al. (71); Guerrero-Peral (72); Ng et al. (73); Owens et al. (74); Pompili et al. (75); Smith et al. (76); Ye et al. (77)	O’Connor (24)	<18–85+	Cancer (prostate, lung, breast); Migraine; Age-related cataract	Self-report questionnaire	In-person or remote	Self-administered without supervision or administered by examiner
Adult Decision-making Competence Scale (A-DMC)	6	Hoffman et al. (78); Matthews et al. (79); Merillat and Gonzalez-Vallejo (80); Peng et al. (81); Szanto et al. (82); Weller et al. (83)	Bruine de Bruin et al. (16)	18–85+	Multiple sclerosis; Depression; Dieters	Performance-based	In-person or remote	Self-administered with or without supervision
Capacity to Consent to Treatment Instrument (CCTI)	4	Fowler et al. (29); Gerstenecker et al. (84); Gerstenecker et al. (85); Parmigiani et al. (52)	Marson et al. (13)	18–85+	Mild cognitive impairment; Alzheimer’s disease dementia; Supranuclear Palsy; Parkinson’s disease; Metastatic cancer	Semi-structured interview w/vignettes	In-person	Administered by examiner
Decision-Making Self-Efficacy Scale (DM-SES)	3	Chen et al. (86); Guo et al. (87); Tsai et al. (88)	Bunn and O’Connor (25)	18–84	Lumbar degenerative disease; Age-related cataract and macular degeneration	Self-report questionnaire	In-person	Self-administered with or without supervision
Mental incapacity assessed using the relevant criteria in the Assisted Decision-Making (Capacity) Act 2015 (Ireland)	2	Curley et al. (39, 40); Murphy et al. (47)	Assisted Decision-Making (Capacity) Act (2015)	18–85+	n/a	Semi-structured interview	In-person	Administered by examiner
Short Portable Assessment of Capacity for Everyday Decision Making	1	Fenton et al. (14)	Lai and Karlawish (89)	65–84	Mild cognitive impairment	Semi-structured interview	In-person	Administered by examiner
Decision Outcome Inventory	1	Sobkow et al. (90)	Bruine de Bruin et al. (16)	18–64	n/a	Self-report questionnaire	Remote	Self-administered without supervision
Decision-Making Process (DMP)	1	Nakayama et al. (26)	Nakayama et al. (26)	18–84	n/a	Self-report questionnaire	Remote	Self-administered without supervision
Health Decision-Making Problems	1	Thompson et al. (19)	Waters et al. (20)	18–84	n/a	Performance-based	Remote	Self-administered without supervision
Heart Failure Self-Management Decision-Making	1	Edmiston et al. (91)	Xu et al. (21)	45–85+	Advanced heart failure	Performance-based w/vignettes	In-person	Administered by examiner
Independent Living Scale (ILS)	1	Emmert et al. (92)	Loeb (18)	45–85+	Dementia	Performance-based	In-person	Administered by examiner
Linguistic Instrument for Medical Decision-Making (LIMD)	1	Stormoen et al. (93)	Tallberg et al. (22)	45–84	Mild cognitive impairment; Alzheimer’s disease dementia	Performance-based w/vignettes	In-person	Administered by examiner
Self-Regulation Questionnaire (SRQ)	1	Kooiman et al. (28)	Brown et al. (27)	18–64	n/a	Self-report	Remote	Self-administered with no supervision
Test of Practical Judgment (TOP-J-9)	1	Rabin et al. (94)	Rabin et al. (17)	45–85+	Subjective cognitive decline; mild cognitive impairment; vascular dementia; Alzheimer’s disease dementia; behavioral variant frontotemporal dementia	Performance-based	In-person	Administered by examiner
The Current Medical Decision-Making Capacity Rating (CMDC)	1	Fowler et al. (29)	Fowler et al. (29)	18–84	Metastatic cancer	Self-report	In-person	Self-administered under supervision
The Patient Participation Competence Scale (PPCS)	1	Tang et al. (95)	Liu (23)	18–84	Breast cancer	Performance-based; Self-report	In-person	Administered by examiner

There were 18 measures focusing specifically on assessing HCDM ability (see Table 3) across 64 articles. Most articles indicated administration of measures in English only (54%), 3% only in Spanish, and 3% of articles administered measures in English and another language. Forty-one percent of articles were administered in another language, including 8% Japanese, 6% Italian, 8% Chinese, 3% Dutch, and 3% German. Sample sizes ranged from 20 to over 1,000. Half of the articles included samples only over age 45 (49%). Across all articles, 77% of articles included participants aged 65–84 and 42% included participants aged 85 and older. Most articles included at least one clinical group in the study sample (65%). Of those articles that included clinical groups, the most common clinical populations were dementia (25%), psychiatric disorders (15%), cancer (12%), and mild cognitive impairment (10%). The majority of measures (79%) were administered in-person, and few measures were administered using technology (e.g., computer, iPad; 16%). Most measures (65%) were administered by a trained examiner, though some were self-administered with (4%) or without supervision (24%). Though we included reliability and validity information as part of the full-text extraction, the vast majority of articles (>90%) did not report reliability or validity information, precluding examination of psychometric properties across measures.

### 3.2 Semi-structured interviews

The most frequently used measure of HCDM ability (*n* = 22 articles) was the MacArthur Competence Assessment Tool for Treatment (MacCAT-T; (12)). The MacCAT-T is a semi-structured interview that is well-validated and has been used across many different clinical populations (see Table 3). The measure takes about 15–20 min and must be administered by a trained examiner. During administration of the MacCAT-T, examinees are given information about a specific medical condition and the risks, benefits, and outcomes of different treatments; the medical condition used is typically one that the examinee has been diagnosed with, but some studies have used standardized scenarios. The examiner then asks questions to assess the examinee’s understanding, appreciation, reasoning, and expression of a choice regarding the specific situation. Several articles also included the Capacity to Consent to Treatment Instrument (CCTI; (13)), which is similar to the MacCAT-T in structure and administration but employs standardized vignettes about a brain tumor and heart blockage to elicit understanding, appreciation, reasoning, and expression of a choice. One article utilized the Short Portable Assessment of Capacity for Everyday Decision Making (SPACED; (14); adapted from (7)), which is akin to the above semi-structured measures but is not focused specifically on healthcare scenarios. Examinees are given a scenario depicting an everyday problem with two possible response options; they then are asked a series of standardized questions designed to assess understanding and appreciation of the situation, as well as to compare between the response options.

### 3.3 Performance-based measures

The next most frequently used type of measure was performance-based tasks. Specifically, 12 articles used the Decision-Making Competence Assessment Tool (DMCAT; (15)), which includes two subscales (i.e., HCDM; financial DM). The healthcare-related items ask respondents to weigh various characteristics of health maintenance organization (HMO) plans; each item has a correct answer that can be identified using deductive reasoning. The next most frequently used performance-based measure was the Adult Decision-making Competence Scale (A-DMC; (16)), which contains items that fit into several different scales (e.g., Resistance to Framing, Consistency in Risk Perception) that assess overall decision-making abilities, not necessarily HCDM in particular. Two measures, the Test of Practical Judgment (TOP-J; (17)) and the Health and Safety subscale of the Independent Living Scale (ILS; (18)), posit scenarios related to HCDM and other DM domains (e.g., what to do if one runs out of a prescription medication while traveling), with specific examiner prompts to generate additional responses if examinees do not initially provide full-credit answers. Examinees’ verbal responses are rated based on completeness and complexity according to scoring guidelines. Several other performance-based measures were identified, though none were used in more than one cited article, including the Health Decision-Making Problems, which consists of four items asking examinees to calculate change in probabilities based on treatment chosen ((19); adapted from (20)); Heart Failure Self-Management Decision-Making, which asks respondents with heart failure how they respond to symptoms that may signal need for medical intervention (21); Linguistic Instrument for Medical Decision-Making, which provides three vignettes and asks examinees to answer a variety of questions that assess comprehension, evaluation, and intelligibility of decision criteria based on linguistic coding (22); and the Patient Participation Competence Scale, which focuses on examinee involvement in DM in terms of communication, information acquisition, emotion management, and autonomous decision-making (23).

### 3.4 Self-report measures

The last category of HCDM measures is self-report. The Decision Self-Efficacy Scale (DSE; (24)) and the Decision-Making Self-Efficacy Scale (DM-SES; (25)) were cited by multiple articles and assess individuals’ confidence in their DM ability (e.g., “I feel confident that I can figure out the choice that best suits me”). Several other self-report measures were identified in the scoping review but were only cited by a single article, including: (1) the Decision Outcome Inventory (16), which lists negative outcomes of poor decision-making (e.g., “Got blisters from sun burn); (2) the Decision-Making Process scale (26), which asks respondents to rate whether they take certain approaches to decision-making (e.g., “I make sure I have all the options available to me”); (3) a modified Self-Regulation Questionnaire (27, 28) assessing self-regulation behavior related to health (e.g., “I can usually find several different possibilities when I want to change something in my health behavior”); and (4) the Current Medical Decision-making Capacity Rating (CMDC; (29)), a self-report measure of perceived independence in understanding, appreciating, reasoning, and expressing health choices (e.g., “Are you able to provide good reasons for whichever treatment choice you made?”).

## 4 Discussion

The current scoping review summarized the extant literature on measures of HCDM used in aging research as part of the larger ARMCADA study, which aims to develop an efficient, comprehensive battery to assess multiple domains of decision-making (DM) ability across the cognitive aging spectrum. Though our review identified many articles that focused on HCDM, only a small number of measures used in these articles were directly relevant to HCDM *ability*, which we have defined as the capacity to make decisions and judgments about one’s health, healthcare, and treatment. The measures that directly assessed HCDM ability fell into three categories: semi-structured interviews using healthcare vignettes, performance-based tasks, and self-report measures.

The most frequently used measure was the MacCAT-T, which exemplifies the current gold standard approach for assessing HCDM ability. The MacCAT-T utilizes a semi-structured interview focused on a medical situation or treatment decision relevant to the individual examinee to elicit evidence of the four components of DM capacity per the Grisso and Appelbaum model (i.e., understanding, appreciation, reasoning, expression of a choice; (6)). This type of measure is thorough, well-established, and adaptable to many different healthcare situations. However, semi-structured interviews are time-intensive (e.g., 20+ minutes) and require administration by a trained examiner who can respond appropriately and effectively to various responses, which limits their utility as screening tools; these measures are not always able to be implemented in situations that require rapid medical decision-making or when there is limited time spent with individuals. Indeed, such interview-based measures are better suited to research or clinical settings with ample time for measure administration; the thoroughness of interviews is also appropriate for clinical situations that require a high level of confidence in the measure’s outcome (e.g., critical treatment decisions).

Performance-based measures like the DMCAT and TOP-J offer more standardization in scoring and pose a lower burden for administration, especially for measures that can be administered with minimal examiner supervision (e.g., multiple choice items). However, measures with discrete correct answers often rely on health literacy (e.g., specialized knowledge of healthcare systems or health insurance), which may be impaired in older adults with cognitive decline even in the absence of a dementia diagnosis (30–32). Over-reliance on health literacy in DM measures also penalizes individuals from historically marginalized groups who are afforded less access to education and healthcare resources. Performance-based measures are also not typically formulated to directly address the four-component model of DM capacity; rather, they are more likely to assess a specific type of reasoning (e.g., deductive reasoning) or characterize an approach to decision-making (e.g., resistance to framing).

Self-report measures of DM ability are the least time- and resource-intensive option, making them the most accessible for inclusion in screening processes. However, the benefit of expediency comes at a cost, as these measures are likely less accurate compared to performance- and interview-based measures and typically are not able to assess different components of DM ability per the four-component model of capacity. One self-report measure, the CMDC (29), which includes specific items meant to assess the four components of DM, showed poor reliability with traditional interview-based measures of the same components, especially in persons with impaired capacity per traditional measures. Indeed, divergence between subjective (i.e., self-report) and objective (i.e., performance-based) measures is a particular concern in older adults with cognitive decline (e.g., (33–35)).

As noted above, though we aimed to identify measures that specifically assessed HCDM *ability*, most articles elicited through our search parameters included measures that fell outside this scope (see Supplementary Table 1). That is, most articles included measures that assessed factors related to healthcare and/or decision-making more broadly (e.g., health literacy, decisional conflict, general health behaviors, treatment satisfaction). These measures provide useful information about the process of making a decision, but do not gauge DM ability per se. Measuring HCDM is inherently complex, and it appears that many studies that are not able to include more formalized measures of DM rely on measures of factors that are peripheral to DM ability, which likely contributes to inconsistency in operationalization of HCDM. Researchers should consider whether selected HCDM measures assess HCDM *ability* or other aspects of the decision process, and effort should be made to balance thoroughness with ease of administration.

Several limitations are worth noting in the current scoping review. First, we only included articles published in the last 5 years, which may have excluded older measures that hold promise for assessing HCDM ability. However, this approach allowed for a focus on the current state of the literature that is likely a close approximation of the measures actively being used in clinical and research settings. Second, whereas we did not exclude studies based on the language of administration of measures, we only included articles that were written/published in English; this choice may have limited identification of innovative measures that have only been described in a different language. Third, we did not include reliability and validity information, in part because this was not the main focus of the review, but also because the vast majority of articles identified in the review did not provide this information. Finally, exclusion of non-peer-reviewed works (e.g., theses, conference abstracts) may have biased the final sample of studies; however, we felt it important to focus on published studies to ensure identification of measures of adequate quality.

Overall, the current scoping review identified several frequently used measures of HCDM, but such measures tend to be time- and resource-intensive. The most frequently used and relevant measures are semi-structured interviews (e.g., MacCAT-T, CCTI) and performance-based tasks (e.g., DMCAT, A-DMC), which require administration by a trained examiner with varying levels of subjectivity in scoring. There is a clear need for a streamlined, easy to administer assessment battery suited to clinical screening and research settings that draws on the strengths of existing measures outlined above to allow for direct assessment of DM ability across the cognitive aging spectrum in line with the well-established four-component model of DM per Grisso and Appelbaum (6). Such a battery could be integrated into clinical practice and thus may allow for early detection of changes associated with age-related neurocognitive decline and help older adults avoid negative health-related outcomes.

## Data Availability

The raw data supporting the conclusions of this article will be made available by the authors without undue reservation.

## Footnotes

^1^ Qualtrics © (2023). The extraction for this paper was generated using Qualtrics software, version XM of Qualtrics. Available at: https://www.qualtrics.com.
