# Supplementary material for: Measures of healthcare decision-making ability in cognitive aging: a scoping review from the Advancing Reliable Measurement in Cognitive Aging and Decision-Making Ability (ARMCADA) research initiative
Source: Front Public Health. 2025 Jun 12;13:1582764. doi: 10.3389/fpubh.2025.1582764 (PMC12198135; doi:10.3389/fpubh.2025.1582764)
Supplement: Supplementary file 1 [file Table_1.docx]

Supplemental Table 1. Measures identified in the scoping review related to health and medical decision-making, excluding measures of healthcare decision-making ability

| Measure category | Measure Name | Original Citation | # Citations* |
| --- | --- | --- | --- |
| **Feelings/attitudes about decision-making** | Decisional Conflict Scale (DCS) | O’Connor, A. M. (1995). Validation of a Decisional Conflict Scale. Medical Decision Making, 15(1), 25–30. https://doi.org/10.1177/0272989x9501500105 | 56 |
|  | Decision Regret Scale (DRS) | Brehaut, J. C., O’Connor, A. M., Wood, T. J., Hack, T. F., Siminoff, L., Gordon, E., & Feldman-Stewart, D. (2003). Validation of a Decision Regret Scale. Medical Decision Making, 23(4), 281–292. https://doi.org/10.1177/0272989x03256005 | 24 |
|  | SURE Scale (DCS-SF) | Légaré, F., Kearing, S., Clay, K., Gagnon, S., D’Amours, D., Rousseau, M., & O’Connor, A. (2010). Are you SURE?: Assessing patient decisional conflict with a 4-item screening test. Canadian family physician, 56(8), e308-e314. | 7 |
|  | Satisfaction with Decision (SWD) Scale | Holmes-Rovner, M., Kroll, J., Schmitt, N., Rovner, D. R., Breer, M. L., Rothert, M. L., Padonu, G., & Talarczyk, G. (1996). Patient Satisfaction with Health Care Decisions: The Satisfaction with Decision Scale. Medical Decision Making, 16(1), 58–64. https://doi.org/10.1177/0272989x9601600114 | 6 |
|  | Decision-making Regret (1-item) | Brodney, S., Valentine, K. D., Vo, H. A., Cosenza, C., Barry, M. J., & Sepucha, K. R. (2022). Measuring shared decision-making in younger and older adults with depression. International Journal for Quality in Health Care, 34(4). https://doi.org/10.1093/intqhc/mzac076 | 2 |
|  | Participation Satisfaction in Medical Decision-making Scale (PSMDS) | Xu, X. L. (2010). The patients' satisfaction with participation in medical decision-making scale: development, reliability and validity. Changsha: Central South University, 1-3. | 2 |
|  | Subjective Decision Quality (SDQ) | Resnicow, K., Abrahamse, P., Tocco, R. S., Hawley, S., Griggs, J., Janz, N., Fagerlin, A., Wilson, A., Ward, K. C., Gabram, S. G., & Katz, S. (2014). Development and psychometric properties of a brief measure of subjective decision quality for breast cancer treatment. BMC Medical Informatics and Decision Making, 14(1). https://doi.org/10.1186/s12911-014-0110-x | 2 |
|  | Combined Outcome Measure for Risk Communication And Treatment Decision-making Effectiveness (COMRADE) Scale | Edwards, A., Elwyn, G., Hood, K., Robling, M., Atwell, C., Holmes-Rovner, M., Kinnersley, P., Houston, H., & Russell, I. (2003). The development of COMRADE—a patient-based outcome measure to evaluate the effectiveness of risk communication and treatment decision making in consultations. Patient Education and Counseling, 50(3), 311–322. https://doi.org/10.1016/s0738-3991(03)00055-7 | 2 |
|  | Decisional Balance (DB) Scale | Plotnikoff, R. C., Blanchard, C., Hotz, S. B., & Rhodes, R. (2001). Validation of the Decisional Balance Scales in the Exercise Domain From the Transtheoretical Model: A Longitudinal Test. Measurement in Physical Education and Exercise Science, 5(4), 191–206. https://doi.org/10.1207/s15327841mpee0504_01 | 1 |
|  | Confidence in Decision-Making | Pérez-Lacasta, M. J., Martínez-Alonso, M., Garcia, M., Sala, M., Perestelo-Pérez, L., Vidal, C., Codern-Bové, N., Feijoo-Cid, M., Toledo-Chávarri, A., Cardona, À., Pons, A., Carles-Lavila, M., & Rue, M. (2019). Effect of information about the benefits and harms of mammography on women’s decision making: The InforMa randomised controlled trial. PLOS ONE, 14(3), e0214057. https://doi.org/10.1371/journal.pone.0214057 | 1 |
|  | Decision Readiness | Tsuda, S., Nakamura, M., Miyachi, J., Matsui, Y., Takagi, M., Ohashi, H., Aoki, S., Ono, H., & Ojima, T. (2019). Decisional Conflict in Home Medical Care in a Family-Oriented Society: Family Members’ Perspectives on Surrogate Decision Making from a Multicenter Cohort Study. Journal of Palliative Medicine, 22(7), 814–822. https://doi.org/10.1089/jpm.2018.0493 | 1 |
|  | Medical Decision-making Regret (1-item) | Yamauchi, K., Nakao, M., & Nakashima, M. (2019). Correlates of regret with treatment decision-making among Japanese women with breast cancer: results of an internet-based cross-sectional survey. BMC Women’s Health, 19(1). https://doi.org/10.1186/s12905-019-0783-5 | 1 |
|  | Decision Satisfaction (1-item) | Rivers, A. S., & Sanford, K. (2021). A special kind of stress: Assessing feelings of decisional distress for breast cancer treatment decisions. Patient Education and Counseling, 104(12), 3038–3044. https://doi.org/10.1016/j.pec.2021.04.020 | 1 |
|  | Satisfaction with the Decision Making Process (SDMP) | Barry, M. J., Cherkin, D. C., YuChiao, C., Fowler, F. J., & Skates, S. (1997). A randomized trial of a multimedia shared decision-making program for men facing a treatment decision for benign prostatic hyperplasia. Disease Management and Clinical Outcomes, 1(1), 5-14. | 1 |
| **Behaviors related to decision-making** | Consideration of Future Consequences Scale (CFC) | Strathman, A., Gleicher, F., Boninger, D. S., & Edwards, C. S. (1994). The consideration of future consequences: Weighing immediate and distant outcomes of behavior. Journal of Personality and Social Psychology, 66(4), 742–752. https://doi.org/10.1037//0022-3514.66.4.742 | 2 |
|  | Emotional Arousal and Decision Strategies Questionnaire | Weller, J. A., King, M. L., Figner, B., & Denburg, N. L. (2019). Information use in risky decision making: Do age differences depend on affective context? Psychology and Aging, 34(7), 1005–1020. https://doi.org/10.1037/pag0000397 | 1 |
|  | General Decision-Making Scale (GDMS) | Scott, S. G., & Bruce, R. A. (1995). Decision-Making Style: The Development and Assessment of a New Measure. Educational and Psychological Measurement, 55(5), 818–831. https://doi.org/10.1177/0013164495055005017 | 1 |
|  | Medical Maximizer-Minimizer Scale (MMS-10) | Scherer, L. D., Caverly, T. J., Burke, J., Zikmund-Fisher, B. J., Kullgren, J. T., Steinley, D., McCarthy, D. M., Roney, M., & Fagerlin, A. (2016). Development of the Medical Maximizer-Minimizer Scale. Health Psychology, 35(11), 1276–1287. https://doi.org/10.1037/hea0000417 | 1 |
|  | Melbourne Decision Making Questionnaire (MDMQ) | Mann, L., Burnett, P., Radford, M., & Ford, S. (1997). The Melbourne decision making questionnaire: an instrument for measuring patterns for coping with decisional conflict. Journal of Behavioral Decision Making, 10(1), 1–19. https://doi.org/10.1002/(sici)1099-0771(199703)10:1<1::aid-bdm242>3.0.co;2-x | 1 |
|  | Deliberation Scale | Wallner, L. P., Li, Y., McLeod, M. C., Hamilton, A. S., Ward, K. C., Veenstra, C. M., An, L. C., Janz, N. K., Katz, S. J., & Hawley, S. T. (2017). Decision‐support networks of women newly diagnosed with breast cancer. Cancer, 123(20), 3895–3903. Portico. https://doi.org/10.1002/cncr.30848 | 2 |
|  | Online Health Information Utilization Questionnaire | Chen, Y.-Y., Li, C.-M., Liang, J.-C., & Tsai, C.-C. (2018). Health Information Obtained From the Internet and Changes in Medical Decision Making: Questionnaire Development and Cross-Sectional Survey. Journal of Medical Internet Research, 20(2), e47. https://doi.org/10.2196/jmir.9370 | 1 |
|  | Problem Solving in Medicine Questionnaire | Chen, Y.-Y., Li, C.-M., Liang, J.-C., & Tsai, C.-C. (2018). Health Information Obtained From the Internet and Changes in Medical Decision Making: Questionnaire Development and Cross-Sectional Survey. Journal of Medical Internet Research, 20(2), e47. https://doi.org/10.2196/jmir.9370 | 1 |
|  | Prostate Cancer Decision-making Questionnaire | Shaverdian, N., Kishan, A. U., Veruttipong, D., Demanes, D. J., Kupelian, P., McCloskey, S., Steinberg, M. L., & King, C. R. (2018). Impact of the Primary Information Source Used for Decision Making on Treatment Perceptions and Regret in Prostate Cancer. American Journal of Clinical Oncology, 41(9), 898–904. https://doi.org/10.1097/coc.0000000000000387 | 1 |
|  | The Stage of Decision-Making Scale (SDMS) | O’Connor, A. User Manual–Stage of Decision Making; Ottawa Hospital Research Institute: Ottawa, ON, Canada, 2000. | 1 |
|  | Vividness of Decision Outcomes | Sobkow, A., Olszewska, A., & Traczyk, J. (2020). Multiple numeric competencies predict decision outcomes beyond fluid intelligence and cognitive reflection. Intelligence, 80, 101452. https://doi.org/10.1016/j.intell.2020.101452 | 1 |
| **Health behaviors and outcomes** | Adherence Determination Questionnaire | DiMatteo, M. R., Hays, R. D., Gritz, E. R., Bastani, R., Crane, L., Elashoff, R., ... & Marcus, A. (1993). Patient adherence to cancer control regimens: Scale development and initial validation. Psychological Assessment, 5(1), 102. | 1 |
|  | The MILES Self-Report Questionnaire | Okonkwo, O. C., Griffith, H. R., Vance, D. E., Marson, D. C., Ball, K. K., & Wadley, V. G. (2009). Awareness of Functional Difficulties in Mild Cognitive Impairment: A Multidomain Assessment Approach. Journal of the American Geriatrics Society, 57(6), 978–984. Portico. https://doi.org/10.1111/j.1532-5415.2009.02261.x | 1 |
|  | Relapse Analogue Task (RAT) | Biernacki, K., Molokotos, E., Han, C., Dillon, D. G., Leventhal, A. M., & Janes, A. C. (2023). Enhanced decision-making in nicotine dependent individuals who abstain: A computational analysis using Hierarchical Drift Diffusion Modeling. Drug and Alcohol Dependence, 250, 110890. https://doi.org/10.1016/j.drugalcdep.2023.110890 | 1 |
|  | Personal Diabetes Questionnaire (PDQ) | Stetson, B., Schlundt, D., Rothschild, C., Floyd, J. E., Rogers, W., & Mokshagundam, S. P. (2011). Development and validation of The Personal Diabetes Questionnaire (PDQ): A measure of diabetes self-care behaviors, perceptions and barriers. Diabetes Research and Clinical Practice, 91(3), 321–332. https://doi.org/10.1016/j.diabres.2010.12.002 | 1 |
|  | Post-fall Decision Category Question | Bergeron, C. D., Friedman, D. B., Spencer, S. M., Miller, S. C., Hilfinger Messias, D. K., & McKeever, R. (2016). An Exploratory Survey of Older Women’s Post-Fall Decisions. Journal of Applied Gerontology, 37(9), 1107–1132. https://doi.org/10.1177/0733464816653361 | 1 |
|  | Self-Care Heart Failure Index (SCHFI) | Riegel, B., Lee, C. S., Dickson, V. V., & Carlson, B. (2009). An Update on the Self-care of Heart Failure Index. Journal of Cardiovascular Nursing, 24(6), 485–497. https://doi.org/10.1097/jcn.0b013e3181b4baa0 | 1 |
|  | Self-Care Decisions Inventory | Page, S. D., Lee, C., Aryal, S., Freedland, K., Stromberg, A., Vellone, E., Westland, H., Wiebe, D. J., Jaarsma, T., & Riegel, B. (2022). Development and testing of an instrument to measure contextual factors influencing self-care decisions among adults with chronic illness. Health and Quality of Life Outcomes, 20(1). https://doi.org/10.1186/s12955-022-01990-2 | 1 |
|  | Unmet Expectations Scale | Wollersheim, B. M., van Stam, M.-A., Bosch, R. J. L. H., Pos, F. J., Tillier, C. N., van der Poel, H. G., & Aaronson, N. K. (2020). Unmet expectations in prostate cancer patients and their association with decision regret. Journal of Cancer Survivorship, 14(5), 731–738. https://doi.org/10.1007/s11764-020-00888-6 | 1 |
|  | ICEpop CAPability Measure for Adults (ICECAP-A) | Al-Janabi, H., N Flynn, T., & Coast, J. (2011). Development of a self-report measure of capability wellbeing for adults: the ICECAP-A. Quality of Life Research, 21(1), 167–176. https://doi.org/10.1007/s11136-011-9927-2 | 1 |
|  | Short Form Health Survey (SF-36) | Ware, J. E., & Sherbourne, C. D. (1992). The MOS 36-ltem Short-Form Health Survey (SF-36). Medical Care, 30(6), 473–483. https://doi.org/10.1097/00005650-199206000-00002 | 1 |
|  | Financial and Health Literacy Scale | James, B. D., Boyle, P. A., Bennett, J. S., & Bennett, D. A. (2012). The Impact of Health and Financial Literacy on Decision Making in Community-Based Older Adults. Gerontology, 58(6), 531–539. Portico. https://doi.org/10.1159/000339094 | 1 |
|  | Health Literacy Instrument for Adults (HELIA) | Tavousi, M., Haeri-Mehrizi, A., Rakhshani, F., Rafiefar, S., Soleymanian, A., Sarbandi, F., Ardestani, M., Ghanbari, S., & Montazeri, A. (2020). Development and validation of a short and easy-to-use instrument for measuring health literacy: the Health Literacy Instrument for Adults (HELIA). BMC Public Health, 20(1). https://doi.org/10.1186/s12889-020-08787-2 | 1 |
|  | Iranian Health Literacy Questionnaire (IHLQ) | Haghdoost, A. A., Rakhshani, F., Aarabi, M., Montazeri, A., Tavousi, M., Solimanian, A., Sarbandi, F., Namdar, H., & Iranpour, A. (2015). Iranian Health Literacy Questionnaire (IHLQ): An Instrument for Measuring Health Literacy in Iran. Iranian Red Crescent Medical Journal, 17(5). https://doi.org/10.5812/ircmj.17(5)2015.25831 | 1 |
|  | Lung Cancer Screening (LCS) Survey | Sakoda, L. C., Meyer, M. A., Chawla, N., Sanchez, M. A., Blatchins, M. A., Nayak, S., San, K., Zin, G. K., & Minowada, G. (2019). Effectiveness of a Patient Education Class to Enhance Knowledge about Lung Cancer Screening: a Quality Improvement Evaluation. Journal of Cancer Education, 35(5), 897–904. https://doi.org/10.1007/s13187-019-01540-3 | 1 |
|  | Over-the-Counter Protection Motivation (OTCPM) Survey | Reddy, A. C., & Chui, M. A. (2024). Using Protection Motivation Theory to develop a survey of over-the-counter decision-making by older adults. Research in Social and Administrative Pharmacy, 20(1), 10–18. https://doi.org/10.1016/j.sapharm.2023.09.002 | 1 |
|  | Rapid Estimate of Adult Literacy in Medicine - Revised (REALM-R) | Murphy, P. W., Davis, T. C., Long, S. W., Jackson, R. H., & Decker, B. C. (1993). Rapid Estimate of Adult Literacy in Medicine (REALM): A Quick Reading Test for Patients. Journal of Reading, 37(2), 124–130. http://www.jstor.org/stable/40033408 | 1 |
|  | Standard Gamble (SG) | Slaughter, K. B., Meyer, E. G., Bambhroliya, A. B., Meeks, J. R., Ahmed, W., Bowry, R., Behrouz, R., Mir, O., Begley, C., Tyson, J. E., Miller, C., Warach, S., Grotta, J. C., McCullough, L. D., Savitz, S. I., & Vahidy, F. S. (2019). Direct Assessment of Health Utilities Using the Standard Gamble Among Patients With Primary Intracerebral Hemorrhage. Circulation: Cardiovascular Quality and Outcomes, 12(9). https://doi.org/10.1161/circoutcomes.119.005606 | 1 |
|  | Geriatric Assessment (GA) | Hurria, A., Gupta, S., Zauderer, M., Zuckerman, E. L., Cohen, H. J., Muss, H., Rodin, M., Panageas, K. S., Holland, J. C., Saltz, L., Kris, M. G., Noy, A., Gomez, J., Jakubowski, A., Hudis, C., & Kornblith, A. B. (2005). Developing a cancer‐specific geriatric assessment. Cancer, 104(9), 1998–2005. Portico. https://doi.org/10.1002/cncr.21422 | 1 |
|  | Clinical Decision Making Involvement and Satisfaction Scale – Patient Version (CDIS) | Slade, M., Jordan, H., Clarke, E., Williams, P., Kaliniecka, H., Arnold, K., Fiorillo, A., Giacco, D., Luciano, M., Égerházi, A., Nagy, M., Krogsgaard Bording, M., Østermark Sørensen, H., Rössler, W., Kawohl, W., & Puschner, B. (2014). The development and evaluation of a five-language multi-perspective standardised measure: clinical decision-making involvement and satisfaction (CDIS). BMC Health Services Research, 14(1). https://doi.org/10.1186/1472-6963-14-323 | 1 |
|  | Treatment Satisfaction Questionnaire for Medication (TSQM) | Atkinson, M.J., Sinha, A., Hass, S.L., Colman, S.S., Kumar, R.N., Brod, M., & Rowland, C. R. (2004). Validation of a general measure of treatment satisfaction, the Treatment Satisfaction Questionnaire for Medication (TSQM), using a national panel study of chronic disease. Health Qual Life Outcomes 2, 12. https://doi.org/10.1186/1477-7525-2-12 | 1 |
|  | Satisfaction with Surgery Outcome (1-item) | Brodney, S., Fowler, F. J., Barry, M. J., Chang, Y., & Sepucha, K. (2019). Comparison of Three Measures of Shared Decision Making: SDM Process_4, CollaboRATE, and SURE Scales. Medical Decision Making, 39(6), 673–680. https://doi.org/10.1177/0272989x19855951 | 1 |
|  | Breast Cancer Surgery Decision Quality Instrument (BCS-DQI) | Sepucha, K. R., Belkora, J. K., Chang, Y., Cosenza, C., Levin, C. A., Moy, B., Partridge, A., & Lee, C. N. (2012). Measuring decision quality: psychometric evaluation of a new instrument for breast cancer surgery. BMC Medical Informatics and Decision Making, 12(1). https://doi.org/10.1186/1472-6947-12-51 | 1 |
|  | Decision-making Participation Self-efficacy Scale (DEPS) | Arora, N. K., Weaver, K. E., Clayman, M. L., Oakley-Girvan, I., & Potosky, A. L. (2009). Physicians’ decision-making style and psychosocial outcomes among cancer survivors. Patient Education and Counseling, 77(3), 404–412. https://doi.org/10.1016/j.pec.2009.10.004 | 1 |
|  | Medical Decision-making Task (MDMT) | Bruce, J. M., Bruce, A. S., Lynch, S., Thelen, J., Lim, S.-L., Smith, J., Catley, D., Reed, D. D., & Jarmolowicz, D. P. (2018). Probability discounting of treatment decisions in multiple sclerosis: associations with disease knowledge, neuropsychiatric status, and adherence. Psychopharmacology, 235(11), 3303–3313. https://doi.org/10.1007/s00213-018-5037-y | 1 |
|  | Medication Use Decision Scale | Ellis, E. M., Klein, W. M. P., Orehek, E., & Ferrer, R. A. (2018). Effects of Emotion on Medical Decisions Involving Tradeoffs. Medical Decision Making, 38(8), 1027–1039. https://doi.org/10.1177/0272989x18806493 | 1 |
|  | Risk Perceptions Scale | Ellis, E. M., Klein, W. M. P., Orehek, E., & Ferrer, R. A. (2018). Effects of Emotion on Medical Decisions Involving Tradeoffs. Medical Decision Making, 38(8), 1027–1039. https://doi.org/10.1177/0272989x18806493 | 1 |
|  | Functional Assessment of Cancer Therapy- Bladder Cancer (FACT-BL) | Chen, X., Wang, W., Xiang, A., & Li, Y. (2020). Long-term functional recovery after orthotopic spiral ileal bladder substitution. Translational Andrology and Urology, 9(2), 665–672. https://doi.org/10.21037/tau.2020.03.16 | 1 |
|  | The Client Evaluation of Self and Treatment (CEST) | Joe, G. W., Broome, K. M., Rowan-Szal, G. A., & Simpson, D. D. (2002). Measuring patient attributes and engagement in treatment. Journal of Substance Abuse Treatment, 22(4), 183–196. https://doi.org/10.1016/s0740-5472(02)00232-5 | 1 |
|  | Treatment Decision-making Survey | McMullen, S., Hess, L. M., Kim, E. S., Levy, B., Mohamed, M., Waterhouse, D., Wozniak, A., Goring, S., Müller, K., Muehlenbein, C., Aggarwal, H., Zhu, Y., Oton, A. B., Ersek, J. L., & Winfree, K. B. (2018). Treatment Decisions for Advanced Non-Squamous Non-Small Cell Lung Cancer: Patient and Physician Perspectives on Maintenance Therapy. The Patient - Patient-Centered Outcomes Research, 12(2), 223–233. https://doi.org/10.1007/s40271-018-0327-3 | 1 |
|  | CAPER TREATMENT Survey | Wilson, L., Zheng, P., Ionova, Y., Denham, A., Yoo, C., Ma, Y., Greco, C. M., Hanmer, J., Williams, D. A., Hassett, A. L., Scheffler, A. W., Valone, F., Mehling, W., Berven, S., Lotz, J., & O’Neill, C. (2023). CAPER: patient preferences to inform nonsurgical treatment of chronic low back pain: a discrete-choice experiment. Pain Medicine, 24(8), 963–973. https://doi.org/10.1093/pm/pnad038 | 1 |
|  | Organ Donation Survey | Liu, C. W., Chen, L. N., Anwar, A., Lu Zhao, B., Lai, C. K. Y., Ng, W. H., Suhitharan, T., Ho, V. K., & Liu, J. C. J. (2021). Comparing organ donation decisions for next-of-kin versus the self: results of a national survey. BMJ Open, 11(11), e051273. https://doi.org/10.1136/bmjopen-2021-051273 | 1 |
| **Capacity to consent to research** | MacArthur Competence Assessment Tool for Clinical Research (MacCAT-CR) | Appelbaum, P. S. and Grisso, T. (2001). MacArthur Competence Assessment Tool for Clinical Research (MacCATCR). Sarasota: Professional Resource Press. | 6 |
|  | University of California, San Diego Brief Assessment of Capacity to Consent (UBACC) | Jeste, D. V., Palmer, B. W., Appelbaum, P. S., Golshan, S., Glorioso, D., Dunn, L. B., Kim, K., Meeks, T., & Kraemer, H. C. (2007). A New Brief Instrument for Assessing Decisional Capacity for Clinical Research. Archives of General Psychiatry, 64(8), 966. https://doi.org/10.1001/archpsyc.64.8.966 | 3 |
|  | 6-item Capacity Assessment Questionnaire | Portley, M., Sherer, C., Wu, T., Farren, J., Danielian, L. E., Scholz, S. W., Traynor, B. J., Ward, M. E., Haselhuhn, T., Snyder, A., & Kwan, J. Y. (2023). Cognitive determinants of decisional capacity in neurodegenerative disorders. Annals of Clinical and Translational Neurology, 10(10), 1816–1823. Portico. https://doi.org/10.1002/acn3.51871 | 1 |
|  | Capacity to Consent to Research Instrument (CCRI) | Marson, D. C., Martin, R. C., Triebel, K. L., & Nabors, L. B. (2010). Capacity to Consent to Research Participation in Adults With Malignant Glioma. Journal of Clinical Oncology, 28(24), 3844–3850. https://doi.org/10.1200/jco.2009.27.9091 | 1 |
| **Shared decision-making** | Control Preferences Scale (CPS) | Degner, L. F., Sloan, J. A., & Venkatesh, P. (1997). The control preferences scale. Canadian Journal of Nursing Research Archive, 21-44. | 4 |
|  | Problem Solving Decision Making (PSDM) Scale | Deber, R. B. (1996). What role do patients wish to play in treatment decision making? Archives of Internal Medicine, 156(13), 1414–1420. https://doi.org/10.1001/archinte.156.13.1414 | 3 |
|  | Involvement in Breast Reconstruction (BR) Decision-making Process Scale | Kuo, N.-T., Kuo, Y.-L., Lai, H.-W., Ko, N.-Y., & Fang, S.-Y. (2018). The influence of partner involvement in the decision-making process on body image and decision regret among women receiving breast reconstruction. Supportive Care in Cancer, 27(5), 1721–1728. https://doi.org/10.1007/s00520-018-4416-6 | 1 |
|  | The Patient Expectation for Participation in Medical Decision-making Scale (PEPMDS) | Xu, X. L., Mao, J., Wang, J., & Zhao, H. (2012). Developing strategy and item selection of the patients' expectation for participation in medical decision making scale. China Mod Med, 19, 162-164. | 1 |
|  | Decision-making Patterns Survey | Yang, L., Song, X., Chen, Y., Li, Y., Gu, Y., Wang, X., Zhu, L., Zhi, M., Ouyang, C., & Guo, H. (2021). Treatment Decision-making in Chinese Inflammatory Bowel Disease Patients. Inflammatory Bowel Diseases, 28(Supplement_2), S76–S84. https://doi.org/10.1093/ibd/izab305 | 1 |
|  | Shared Decision-making Process Scale (4-items) | Nakayama, K., Yonekura, Y., Danya, H., & Hagiwara, K. (2022). COVID-19 Preventive Behaviors and Health Literacy, Information Evaluation, and Decision-making Skills in Japanese Adults: Cross-sectional Survey Study. JMIR Formative Research, 6(1), e34966. https://doi.org/10.2196/34966 | 1 |
| **Proxy decision-making** | Combined Scale for Proxy Informed Consent Decisions (CONCORD scale) | Shepherd, V., Hood, K., Gillies, K., & Wood, F. (2022). Development of a measure to assess the quality of proxy decisions about research participation on behalf of adults lacking capacity to consent: the Combined Scale for Proxy Informed Consent Decisions (CONCORD scale). Trials, 23(1). https://doi.org/10.1186/s13063-022-06787-8 | 1 |
| **Decision-making aids** | Preparation for Decision-Making (PrepDM) | Bennett, C., Graham, I. D., Kristjansson, E., Kearing, S. A., Clay, K. F., & O’Connor, A. M. (2010). Validation of a Preparation for Decision Making scale. Patient Education and Counseling, 78(1), 130–133. https://doi.org/10.1016/j.pec.2009.05.012 | 8 |

Note. Measures of HCDM ability are included in Table 3 in the main manuscript.

*Number of articles citing each measure in the scoping review.
